# An Approach for Modeling and Simulation of Virtual Sensors in Automatic Control Systems Using Game Engines and Machine Learning

**DOI:** 10.3390/s24237610

**Published:** 2024-11-28

**Authors:** João Rosas, Luís Brito Palma, Rui Azevedo Antunes

**Affiliations:** 1 NOVA School of Science and Technology, NOVA University Lisbon, Campus de Caparica, 2829-516 Caparica, Portugal; jrosas@uninova.pt; 2 CTS-Uninova & LASI, Campus de Caparica, 2829-516 Caparica, Portugal; 3 ESTSetúbal, Instituto Politécnico de Setúbal, Estefanilha, 2914-508 Setúbal, Portugal; rui.antunes@estsetubal.ips.pt

**Keywords:** automatic control systems, systems modeling and simulation, systems virtualization, game engines, machine learning, industry 4.0

## Abstract

We live in an era characterized by Society 4.0 and Industry 4.0 where successive innovations that are more or less disruptive are occurring. Within this context, the modeling and simulation of dynamic supervisory and control systems require dealing with more sophistication and complexity, with effects in terms of development errors and higher costs. One of the most difficult aspects of simulating these systems is the handling of vision sensors. The current tools provide these sensors but in a specific and limited way. This paper describes a six-step approach to sensor virtualization. For testing the approach, a simulation platform based on game engines was developed. As contributions, the platform can simulate dynamic systems, including industrial processes with vision sensors. Furthermore, the proposed virtualization approach allows for the modeling of sensors in a systematic way, reducing the complexity and effort required to simulate this type of system.

## 1. Introduction

### 1.1. Motivation and Goals

The control and automation engineering field faces new technological challenges due to the increased complexity of problems, variety of situations, variability of systems, and the resources available for adequate control strategies.

Newer tools that help deal with the growing complexity of problems have also emerged with more innovative and sophisticated features. In addition, new technological paradigms such as Industry 4.0, digital twins, virtual reality, and artificial intelligence, among others, multiply the possibilities in modeling and simulating control systems.

Within this context, the modeling and simulation of dynamic supervisory and control systems require dealing with more sophisticated and complex aspects, leading to higher development costs and more errors. One of the most difficult aspects of simulating these systems is handling vision sensors.

This research work is part of this context, as it contributes to an approach for sensor model creation and simulation inside virtual environments. We aim to provide a systematic methodology for virtualizing these sensors with higher complexity. Specifically, the aim is to verify whether it is possible to replicate and extend the behavior of complex sensors in an agile and reliable way. This is relevant to the realistic simulation of control systems, for example, in the context of monitoring and maintenance and more broadly within digital twin systems.

As proof of concept, we will test the sensor virtualization approach by developing a platform based on game engines. Many aspects of virtualizing physical systems have already been addressed in the state of the art, and there are even specific tools for this. Our approach will address the most relevant aspects of the virtualization process, namely, how to model the structure of a physical system, how to simulate its behavior, how to replicate the operation of actuators, and how to model and replicate sensors with more complex functionality. Although all these aspects are more or less handled, the virtualization of sensors is the main focus of our research work.

Simulating the behavior of sophisticated sensors, such as vision, is more difficult, and requires more complex simulation mechanisms, as illustrated by research works [1,2,3].

As such, we propose a systematic modeling approach based on game engines. The modeling process includes several consistent steps that can be applied to modeling a system in general and to its sensors in particular. The general behavioral features of virtual systems, actuators, and their corresponding effects on them, like movement, are also considered.

As our contribution, we aim to determine how straightforwardly we can simulate the behavior of virtual environments, such as the one proposed as proof of concept. The scenario consists of a fruit distribution line, composed of a conveyor, a fruit splitter, and several cylinders that push the fruit inside the corresponding boxes. A camera located on the top observes the conveyor movement. Three other cameras are used to observe the fruit passing by.

In such a way, we aim to determine how easy it is to obtain a simulation model of a camera to measure the speed of a conveyor belt. We also aim to determine how straightforward it is to use cameras to observe the fruit passing by and be able to classify whether they are apples, oranges, or pears. In order to recognize the fruit, the images obtained are made available to a classifier based on a convolutional neural network (CNN or ConvNet) that has been trained for this purpose.

As a way to approach the proof-of-concept system in a more realistic standing, the fruit distribution line is controlled by a virtual programmable logic controller (PLC), which will coordinate the movement of the conveyor and the cylinders. The PLC will also control the speed of the conveyor belt using a PID controller. Since everything will occur in a virtualized environment, the PLC is also a simulated replica of a real PLC. The PLC is not the focus of our research; it only implements the functions needed to control the conveyor, namely, PID control, the set and reset operators, and the Time Pulse timer.

Therefore, the final result is the formulation of a systematic approach to model the behavior of virtual sensors. As such, the proposed approach does not intend to compete with other simulation platforms, such as Factory I/O [4] or CoppeliaSim [5]. The research work in [6] analyzes and compares the characteristics of various platforms in some detail, although the study does not include vision sensors.

### 1.2. Paper Structure

This paper is structured as follows: Section 2 comprises a state-of-the-art literature review, relevant concepts, and related research. Section 3 describes our systematic approach to virtualizing sensors in the context of virtual environments using game engines. Section 4 describes the tests to validate the system and then reflects on the results. Section 5 includes conclusions and future work.

## 2. Literature Review and Relevant Concepts

Before starting the literature review, it is relevant to justify the order of the sections. The research work is multi-disciplinary, involving concepts from various areas. As such, we organized the multiple sections so that topics mentioned in one section were already considered in previous ones. We begin with a brief introduction to modeling and simulation in the context of Industry 4.0. Then, we proceed to introduce game engines as a way to implement virtual environments. Then, we address the issue of developing models for virtual environments supported by game engines. We then present a comparative table of the various features and functionalities of the analyzed simulators, including the proposed simulator. The related research is aimed at identifying gaps.

### 2.1. Modeling and Simulation in the Context of Industry 4.0

The fourth industrial revolution, Industry 4.0, started around 2011 and was based on intelligent manufacturing, which was supported in factories using emerging technologies of automation, control, information, and communication [7,8,9,10,11,12]. The main objective is maximizing efficiency, quality, and productivity while reducing energy consumption and pollution. The main drivers of Industry 4.0 include several concepts and technologies that transform industrial production and some sectors of society. Here are some of the most important ones in which the modeling and simulation of complex dynamic systems play an essential role:

1. Cyber–Physical Systems (CPSs);
2. Internet of Things (IoT) and Internet of Industrial Things (IIoT);
3. Cloud Computing;
4. Artificial intelligence (AI);
5. Big data and Data Analysis;
6. Additive Manufacturing (3D Printing);
7. Virtual and Augmented Reality (VAR);
8. Autonomous Robotics.

Complex dynamic system modeling and simulation play a crucial role in the design of industrial systems in the context of Industry 4.0 for several reasons [13,14,15]:

1. Validation and Verification: before implementation, it is possible to simulate, validate, and verify that the designed system meets all specifications; this helps to identify and correct potential faults, failures, or inefficiencies in advance;
2. Cost and Risk Reduction: modeling and simulation allow for testing different scenarios and solutions without the risks and costs associated with real-world experimentation; this is especially important in complex and expensive industrial systems;
3. Performance Optimization: through modeling and simulation, it is possible to optimize system performance and behavior, adjusting parameters and configuring devices and components to achieve maximum efficiency and productivity;
4. Integration of Advanced Methodologies and Technologies: this facilitates the integration of advanced methodologies and technologies such as artificial intelligence, big data, and IoT, creating a more intelligent and more connected production industrial environment;
5. Decision-Making Support: this provides a solid basis for making informed decisions, allowing managers to evaluate the impact of different strategies and choose the best approach to achieving business goals;
6. Adaptability and Flexibility: allows for the creation of more adaptable and flexible industrial systems capable of responding quickly to changes in the global market or operational conditions.

Digital twins, being models (replicas) of physical systems, processes, mechanisms, or objects, need to use virtual graphical environments to emulate and replicate the behavior of real systems [16,17,18,19,20], and they are essential for achieving the main goals of Industry 4.0:

1. Flexibility, Agility, and Increased Productivity;
2. Enhanced Product Quality and Cost Reduction;
3. Real-Time Decision Making;
4. Customization and Personalization;
5. Innovation, Competitiveness, and Sustainability.

The combination of the drivers mentioned above, the increasing complexity of dynamic systems, and interconnectivity leads to new challenges in the modeling and simulation of supervision, automation, and control systems. New problems and research questions are identified, including sensors’ virtualization and use in sophisticated virtualized simulation environments.

### 2.2. Game Engines

Game engines are part of a very complex area of technology, so we will briefly explain the concept and the relevant aspects of their functionality. For a more in-depth and comprehensive description of game engines, see [8].

The use of game engines in research was proposed in [21]. Their application in control system simulation was proposed in [13,14,22,23]. A game engine can be seen as a software development platform used to help create digital games. Most widely used game engines are Unity [24], Unreal Engine [25], Cry Engine [26], GoDot [27], and GameMaker Studio [28].

Game engines provide numerous tools to help develop the crucial phases of a game. These tools are as follows:

1. Graphic rendering for creating 2D/3D graphics, which includes textures, shadows, lighting, and other visual effects;
2. The physics engine, which allows for simulating the laws of physics that can be applied to objects in a game, namely, movement, forces, gravity, collisions, and friction, among others;
3. Collision systems, which model and define events when collisions between objects occur;
4. The audio system, which allows for playing sound, music, and other sound effects;
5. The animation engine, which allows for performing complex animations on the objects in a game, using the multiple animation techniques available (scripted, rigged, etc.);
6. The mechanism for the behavior of game objects can be performed using programming languages, such as C++, C#, Java, Python, and visual scripting.

**Figure 1:**
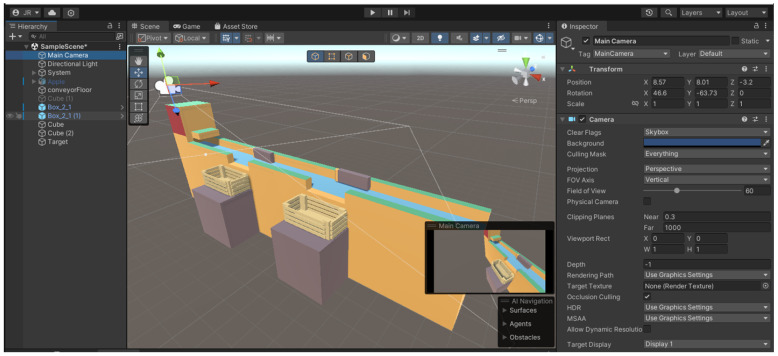
Unity development environment.

A game comprises a set of scenes, each of which represents several game levels, including the presentation and the configuration menus. Figure 1 shows the environment for creating a game using Unity.

The left-hand side represents one of the game’s scenarios, which is organized hierarchically since, typically, game objects can be children of other game objects. The middle window is where the game scene is rendered. The window on the right shows the components and corresponding properties of the various objects comprising the scene.

The main components of a game object are the transform (for defining position, orientation, and scaling) and the mesh renderer used to render the object into a 3D world. Other components that can be added to objects are colliders, the rigid body, animators, audio sources, scripts for their operation, etc.

**Figure 2:**
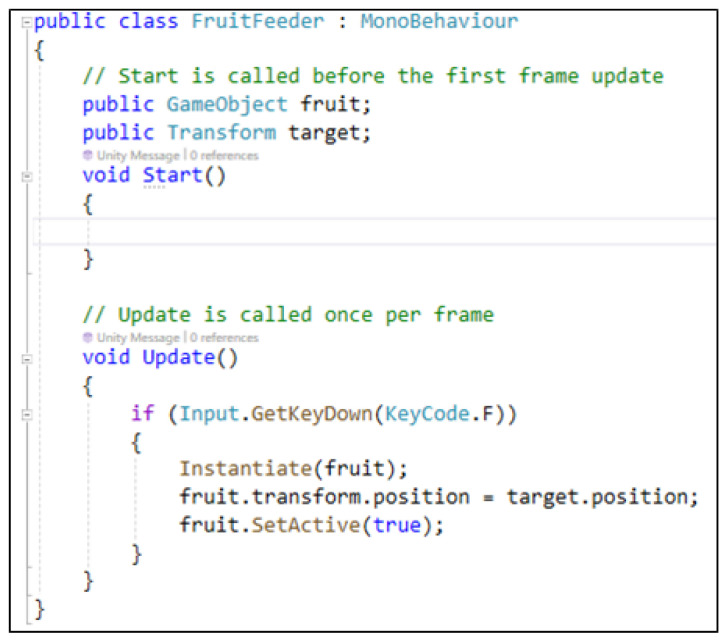
An example of a class for establishing the behavior of a game object in Unity.

The behavior of the game objects is developed with scripts using specific programming languages. For example, Figure 2 shows the C# script for programming an object within Unity.

### 2.3. Modeling Virtual Environments

Modeling a system within a virtual environment is characterized by its significant complexity. As our research focuses on sensors, we will approach modeling in virtual environments in a broad, general way, giving more emphasis to sensors.

Within our approach, the concept of virtualizing a sensor (making one version that does more than its physical equivalent) is extended to consider the aspects of modeling in virtual environments, including the ability to interoperate with other systems integrated into the virtual environment.

System virtualization is a process that involves creating a model that faithfully represents the structure and behavior of an existing tangible or intangible system. Among other roles, this model can simulate the system’s behavior in relevant situations or scenarios. These models are then used to simulate behavior within virtual environments in various situations, mainly when it reacts to events or commands to coordinate their operation.

The concept of abstraction is closely related with the construction of models. Abstraction means considering only the relevant aspects of a system and deliberately ignoring aspects that are not relevant, i.e., do not contribute to the objectives or interests in focus. The complexity and level of granularity in modeling and virtualizing a system can be as high as possible. In practical terms, this effort is made up to a level still relevant to the objectives. In our approach, and to test the system in more complex scenarios, one of the most sophisticated components simulated was the construction of a replica of an industrial PLC.

As mentioned before, the main focus of this work is the virtualization of sensors in the context of Industry 4.0 virtual environments. A critical and challenging aspect of virtualizing is the perception components of systems, especially very complex ones, which vary according to the types of sensors being considered.

The simulation of contact sensors is relatively straightforward with game engines. The approach is to detect the collision between two objects by handling the event of the corresponding colliders. Proximity sensors can be modeled by determining the distance between the sensor and a reference object. However, in more complex cases, more sophisticated approaches are required. For example, the simulation of a distance sensor (ultrasound and LiDAR) can be performed using a ray because when it intercepts an object, the respective distance can be obtained [29]. Visual sensors are more complex to model and simulate as it involves several stages, from capturing images, processing, and providing them to classification models.

### 2.4. Comparative Analysis of Simulators

In Table 1, a comparative analysis of the main features for different simulators is presented. In this table, our simulator is compared to three relevant commercial products: (a) Factory I/O, https://factoryio.com/, accessed on 15 November 2024; (b) Machines Simulator, https://www.nirtec.com/easyplc-machines-simulator/, accessed on 15 November 2024; and (c) CodeSys, https://www.codesys.com/, accessed on 15 November 2024. Our simulator can be extended with more components developed in Python and C#. One interesting feature is that our free simulator can be programmed in the Python language, and used to model and simulate complex automation and control systems, in a local or a distributed environment, exploring a wide range of scenarios.

### 2.5. Related Research

#### 2.5.1. Virtual Environments

The main focus of the related work is to determine how virtual sensors have been approached in other current research works, namely, regarding their agile integration in virtual environments. Other aspects, like control and automation systems, simulation, and modeling tools, will be addressed more generally.

In general, modeling systems for virtual environments have revealed some challenges, considering such types as digital twins (DTs). There is still a lack of consensus on the definition and components of digital twins, making it difficult to standardize them. Furthermore, there is no broadly accepted systematic and comprehensive approach to define and model systems, their physical properties, virtual models, and their interoperability aspects [30], among others. This includes the existence of a variety of DT models specific to each area.

There are also some challenges regarding the fidelity and realism of the models. These models are only helpful if they can realistically replicate natural systems’ physical and dynamic properties and behaviors. This issue requires the rigorous incorporation of physical geometric, dynamics, and behavioral information into virtual models, including sensory components that require complex, large-scale processing but whose integration in a standard way to maintain their fidelity has not yet been fully resolved [31].

Another challenge is the autonomy and adaptability of these environments. These systems must be able to make decisions autonomously and in real time, which requires further research, especially regarding AI/ML models [30]. There are still technical challenges in integrating the different technologies (AI/ML, big data, IoT, etc.) needed in sophisticated virtual environments [30].

An emerging challenge is the creation of customized DT systems in specific areas (health, manufacturing, etc.), as each area has its own requirements that may require customized DT/virtualized models [31].

Currently, research efforts include the development of common standards for modeling virtual environments and digitizing their components. One such example is the Asset Administration Shell (AAS) [32] within the framework of Industry 4.0. The AAS allows for the use of a standard approach for developing standard, modular, extensible, and interoperable simulation models for virtual environments, such as DTs.

#### 2.5.2. Virtual Sensors

Virtual sensors are software-based abstractions that replace real sensors and can extend, improve, and rectify their operation [11,25,26,33,34]. Virtual sensors produce signals that are the fusion of other physical or virtual sensors [35,36]. An example of sensor fusion is when each building floor has several temperature sensors, but a virtual sensor aggregates them, providing a single temperature measurement for each floor [37]. Another advantage of virtual sensors is that they are easier to share with different systems, unlike physical sensors, which typically have more restricted access [38]. Usually, these sensors are available as services, following the Sensor-as-a-Service paradigm [39], implemented and made available on Cloud platforms [37].

From a digital twin perspective, a virtual sensor is a software-based device that can imitate the structure and replicate the behavior of a real sensor within a virtual environment. As stated by [40], “a virtual sensor is a system predicting a variable, based on connections between other variables in the system, not direct measurement”.

Another advantage of virtual sensors is that they allow for the collection of synthetic data from virtual environments. For example, the study described in [41], developed within the context of COVID-19, proposes using virtual sensors in a virtual environment to observe and measure the social distance between 3D representations of people, collecting synthetic data to train and validate neural networks.

Several authors have already proposed the creation of sensors with complex functionality within virtual environments, mainly using game engines [31,32].

In short, the existing research work is already focused on creating complex sensors for virtual environments. The gap identified is the lack of a systematic and straightforward approach to the virtualization process, which is one of our main contributions.

## 3. The Proposed Virtualization Approach

### 3.1. Conceptual Architecture

**Figure 3:**
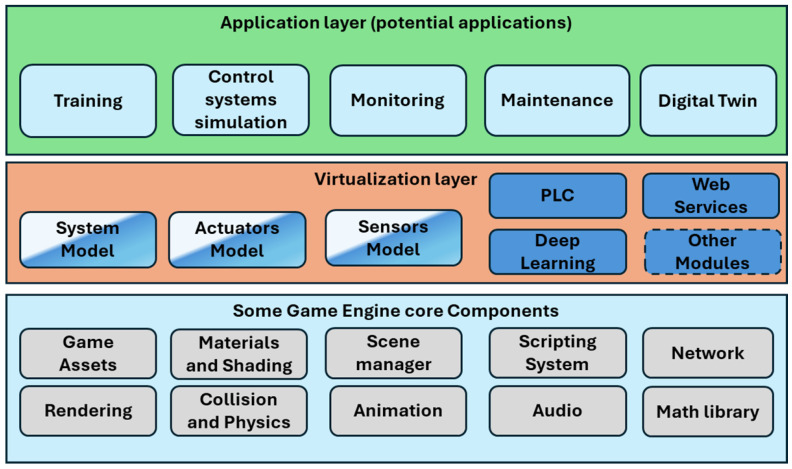
The proposed conceptual architecture.

The starting point for describing our approach to modeling sensors in virtual environments requires a conceptual architecture to highlight the main components relevant to the virtualization process. Figure 3 shows the main modules of the architecture. The lowest layer represents the blocks of the architecture that are internal to the game engine used.

The intermediate layer contains the blocks responsible for the virtualization process and will be our research’s focus. The system model component is responsible for representing the state of the virtual system at each instant. The “actuator model” block is responsible for characterizing and defining the actuator behavior in the virtual environment. The “sensor model” block describes the properties and behavior of each sensor within the virtual environment. The PLC block represents the virtual programmable logic controller. The web services block allows for interconnection between the virtualization components and the layers below the game engine. This block, therefore, deals with the interoperability aspects of the platform. The blue blocks represent fully developed Python components and are available via web services. The blocks with two colors represent partially developed components within the game engine (C#) and partially in the virtualization layer (Python). The behavior of the system being simulated is represented by state variables. The values of these variables are sent to the back-end part of the architecture as described below and stored in a state variable dictionary that is available to the components of the platform. The intermediate virtualization layer can also incorporate other blocks or components. For example, we could integrate a Petri net executor or GRAFCET as an alternative to the PLC.

The topmost layer typically represents the application layer, where scenarios are implemented and made available for specific virtual environment applications. In the cases shown in Figure 3, we have, for example, training activities, the simulation of control systems, the monitoring of manufacturing systems, use in maintenance, and the implementation of digital twins.

**Figure 4:**
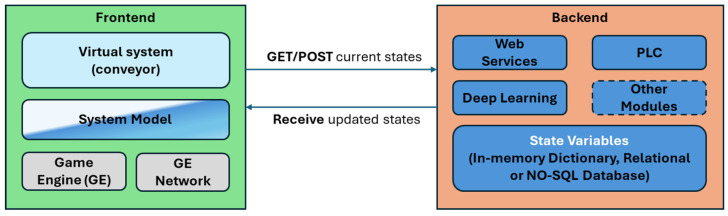
Architectural aspects and interactions in the proposed architecture.

Figure 4 shows another architectural aspect describing how the elements are organized and interact in the proposed platform, namely, their distribution between the front-end and back-end.

The front-end side of the virtual environment provides and renders the system being simulated and allows for interaction with human users. It can be deployed in a web application (Web GL) or native application (Windows, Android, etc.). The back-end side contains the components that support the virtual environment life-cycle and allows for the interoperability of various components via web services. The PLC module is included in the back-end and could be an external element, accessible though a communication protocol, such as Modbus. Similarly, other elements, such as ML models, could be integrated or be clients of the back-end via their correspondent APIs.

**Figure 5:**
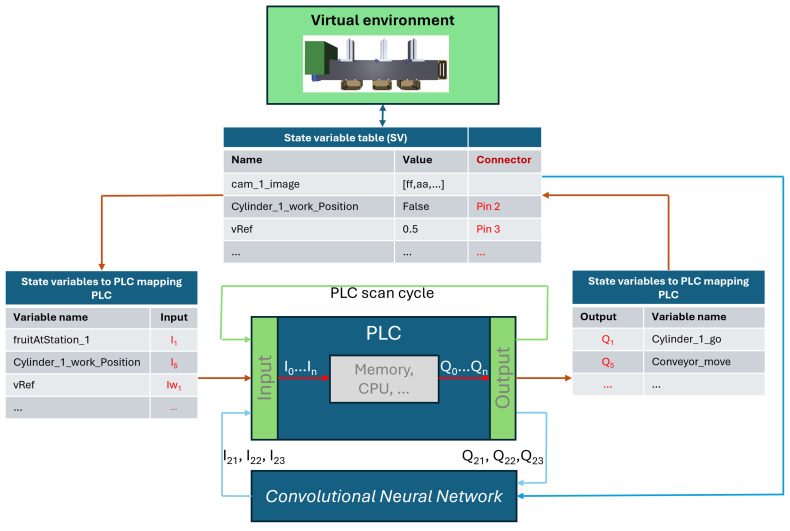
Illustration of the state variables flows between the several components of the platform.

In addition to the architectural aspects presented, it is also necessary to highlight the interactions between the various components, which are illustrated in Figure 5. The figure illustrates that the system’s overall states are represented using state variables. These variables are periodically sent to the server and saved as shareable memory between the architecture components, namely, in the mentioned state variable dictionary. The values of these variables are then transferred to other variables, for instance, when establishing the wiring between the virtual system and the PLC.

The PLC’s input variables are used by the program loaded into its memory to make the control decisions. These decisions are placed in the variables representing the PLC’s output channels. The output variables are then converted into state variables that define the system states operating in the virtual system. Based on the new values of these variables, the system, with the help of the game engine, starts to behave according to their respective values, for example, moving the conveyor or one of the cylinders forward.

As described, the proposed approach is intrinsically simple and easy to replicate for other situations or environments. One of the main attributes of the proposed approach is its simplicity.

### 3.2. Sensor Virtualization

One of the gaps and challenges identified in the state of the art was the existence of approaches for obtaining models for virtual sensors, mainly those that were complex or too specific to the respective areas. As stated before, our contribution consists of proposing a simple and systematic approach to the virtualization of simulation models for virtualized environments and sensors, especially those with more complex functionality. The proposed approach is composed of six main steps. We will use game engine components to show how it can be implemented.

**Figure 6:**
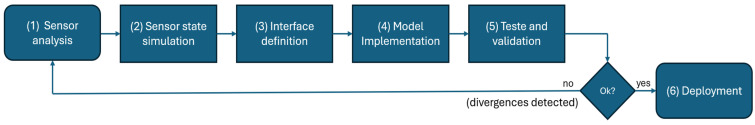
Suggested sensor virtualization process.

In this sense, virtualization consists of creating a mechanism (software or service) to model a physical sensor’s behavior, allowing it to behave indistinguishably from its real counterpart. In Figure 6, the virtualization process is depicted, and the respective steps are an adaptation of design and engineering processes that occur in other areas, namely, software engineering [42], control and automation [43], and sensor simulation [44], among others.

Specifically, the functionality of each of these blocks is as follows:

1. It allows for analyzing the relevant physical characteristics and behavior to be virtualized and simulated. The aim is to determine the sensor’s data types, signal frequencies, and operating conditions that will be simulated within the virtual environment.
2. It consists of specifying the internal process of the virtual sensor to simulate the states, variables, or data generated by the sensor. It can be performed through mathematical models, simulation algorithms, or even recorded data mimicking real sensor data.
3. It allows for defining the virtualization interfaces that mimic the channels or other communication methods that the real sensor uses. Virtual digital and analog connectors can be defined, and web or broadcasting services can be determined based on the sensor’s state variables.
4. The implementation of the virtual sensor takes the form of a software module or service, which initiates and coordinates the operation of the simulation process specified in the previous step. Additionally, it will allow the module to interact with other system components.
5. To guarantee its reliability, rigorous validation tests are required to ensure that the virtual sensor can provide reliable data and function consistently under simulated conditions. Supposing there is a divergence between the actual and expected behavior of the sensor, the differences detected are fed back into step 1 so that the specification can be rectified and the construction of the virtual sensor changed.
6. The last step consists of integrating the sensor into the virtual environment under consideration. From here, the sensor can also be used by other virtual environment components via the interfaces defined in step 3.

As mentioned before, the virtualization of sensors using game engines is an approach that has already been used in other works.

Having already established the behavior model and the sensor interfaces, the most critical step in virtualization in a game engine is step 4 in the proposed approach in the previous section. Therefore, it is essential to illustrate how each type of sensor can be implemented within a game engine, as shown in Table 2. This table is not meant to be exhaustive but to illustrate how to implement the approach with a game engine.

### 3.3. Proof-of-Concept Process

**Figure 7:**
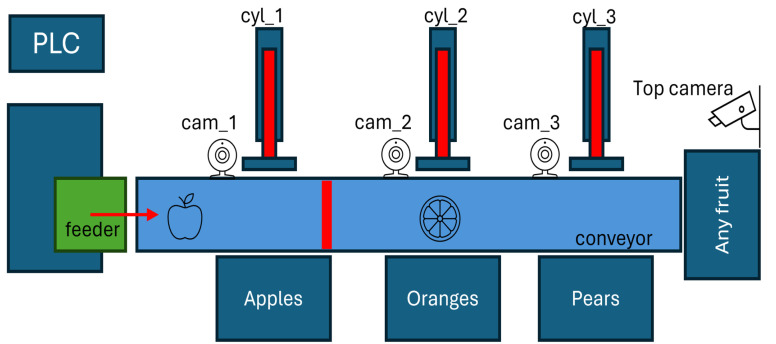
Fruit splitter proof of concept.

To test the concepts presented in the previous sections, we will describe a scenario that constitutes a proof of concept for the proposed approach. The process consists of a fruit sorting line comprising an input feeder, a conveyor belt coupled to a motor, and three pneumatic cylinders that push the fruit into the respective boxes. This system is illustrated in Figure 7.

The feeder periodically places a piece of fruit, apple, pear, or orange, on top of the conveyor belt. The conveyor transports the fruit to the right. A PID controller will control the speed of the conveyor, running inside a virtual PLC, as described in a further section. This controller receives the current speed of the conveyor and compares it with a reference speed, thereby determining the power required by the motor to move the conveyor. A red strip on the carpet of the conveyor and a camera on the top of the system are used to measure the speed at which the conveyor is moving.

The first step in developing a virtual version of this system was to build its structure inside a game engine, in this case, Unity. Since a high-definition, realistic visualization was not required, the system was developed using only geometric primitives. The non-primitive elements were obtained from the Asset Store repository, which provides paid and free assets [45].

**Figure 8:**
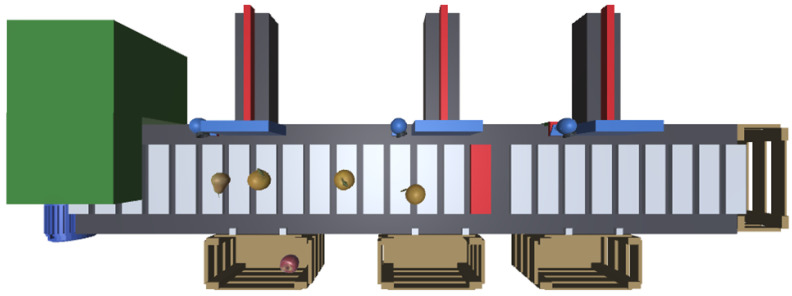
Development of the proof of concept with Unity.

In Figure 8, the overall system can be observed. The fruit was also obtained from the mentioned repository. As the figure shows, the 2D schema presented before (Figure 7) resulted in a satisfactory corresponding 3D implementation. The fruit assets are freely available in the unity Asset Store [46], as well as the boxes [47].

### 3.4. Actuator Modeling

Although it is not the focus of this work, it is important to briefly describe how the behavior of the actuators is implemented within the virtual environment. Since a game engine is being used, their behavior will be materialized using the components of the objects defined within the game scenario. Typically, the activation effect of an actuator manifests itself through the movement of some game objects representing mechanical or physical devices. Each effect or movement depends on the value of a given state variable. When the value changes, there is eventually a movement or animation effect, namely, linear, circular, or other.

**Figure 9:**
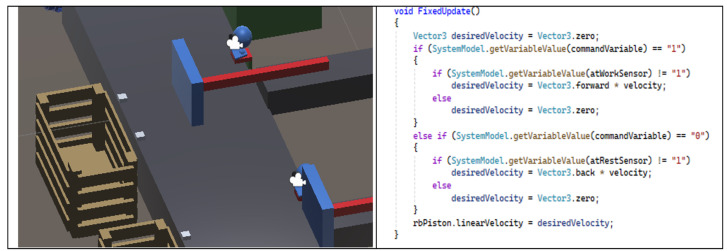
Actuator modeling with Unity.

Figure 9 illustrates the effect of the actuator on one of the pneumatic cylinders in the fruit dispenser. When the state variable changes to the value “1”, it starts to move as established in the C# script shown in the figure. The cylinder stops when it reaches the working limit.

### 3.5. Sensor Modeling

#### 3.5.1. Proximity Sensor Modeling

In this section we describe how to apply the proposed sensor virtualization approach to obtain the models in order to make our virtual environment similar to the fruit distribution station described above. To make the instantiation of the approach clear, let us start with the most straightforward sensor, the proximity sensor, which provides Boolean values. Table 3 shows the application of the method for this sensor from step 1 to step 6.

**Figure 10:**
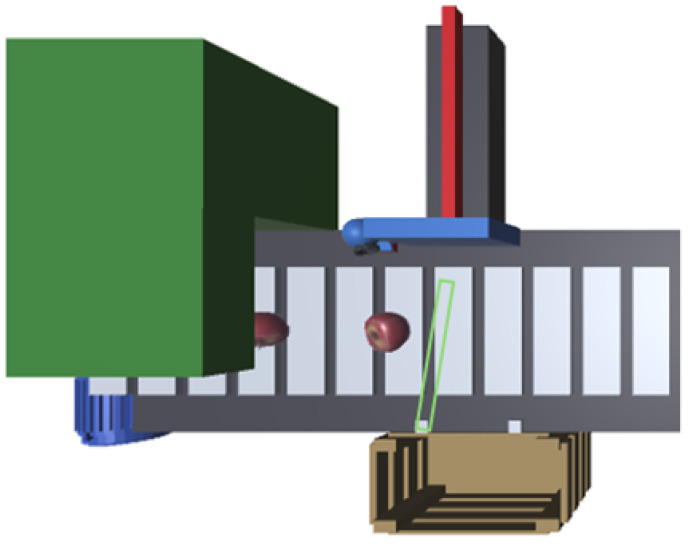
An example of modeling a proximity sensor using a collider.

**Figure 11:**
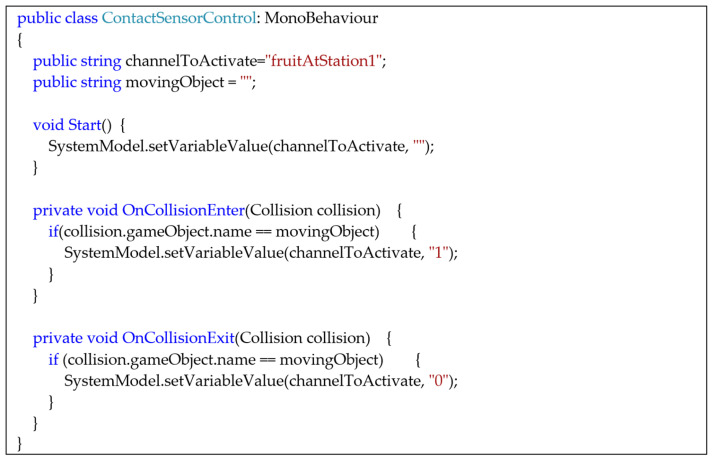
The C# script that handles the sensor state.

Figure 10 shows the binary presence sensor next to the apple collection station. The green rectangle above the conveyor belt represents the collider associated with the sensor’s operation. Figure 11 presents the C# script for detecting the fruit and assigning the corresponding value to the state variable. When the collider to the incoming object, e.g., the fruit, intercepts the sensor collider, the *OnCollisionEnter* handler event is executed.

#### 3.5.2. Sensor for Measuring the Conveyor Belt Speed

One of the aspects mentioned as a contribution of this work was to find out to what extent a complex and sophisticated sensor could be modeled. For achieving this goal, we are going to use a camera to measure the speed of the conveyor belt.

As such, a camera above the fruit separator observes the carpet’s movement, specifically the red strip (Figure 8). The camera’s recognition system periodically receives images from the camera to detect the strip and determine its successive position over time to measure the speed. This process was a little challenging, as it involved collecting images from a virtual camera that is used to observe artificial objects moving in a virtual environment inside a game engine.

Finally, to estimate the conveyor speed, the successive images captured are sent to a Python module that uses OpenCV [48] to identify the red strip and compute its position from the left side of the image. In Table 4, the virtualization process of this task is described.

#### 3.5.3. Fruit Identification Sensors

**Figure 12:**
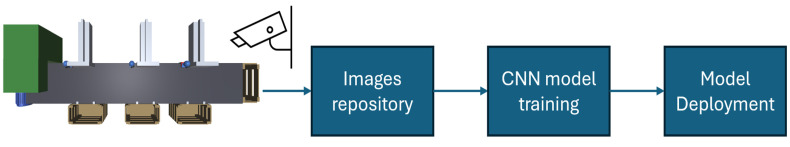
The pipeline for the CNN ML process.

As mentioned before, three cameras were used to detect and recognize the fruit. As the conveyor moves, the fruit passes in front of the cameras. At that point, the proximity sensor is activated. Triggering this sensor will in turn trigger the capture of an image from the camera observing the fruit. This image is sent to the classification module, which uses a convolutional neural network (CNN) to identify the fruit. The modeling and training pipeline of the network is typical in ML studies, as illustrated in Figure 12. According to the proposed six-step approach, this is performed as shown in Table 5.

The fundamental difference with the current Machine Learning pipelines is that the training images, in this case, the fruit coming out of the feeder, are obtained from a virtual camera, which watches the fruit passing through a virtual environment. The trained network is then deployed on the platform’s back-end, and its use is initiated via the virtual PLC’s output channels.

**Figure 13:**
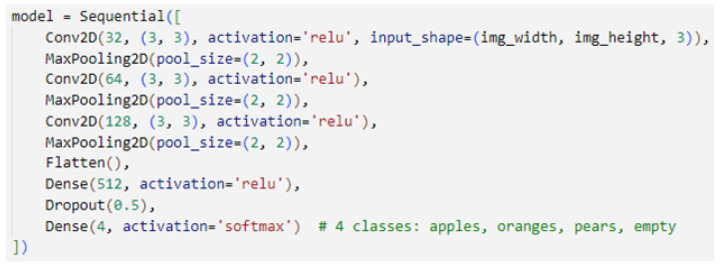
Modeling the CNN network using Keras in Python.

The ConvNet’s structure is described in Figure 13. The network was created with the Keras Sequential API. The network consists of several convolutional and pooling layers, a flattened layer, a dense layer, a dropout layer, and a dense output layer. For a detailed explanation of the structure of ConvNet networks, see [49].

**Figure 14:**
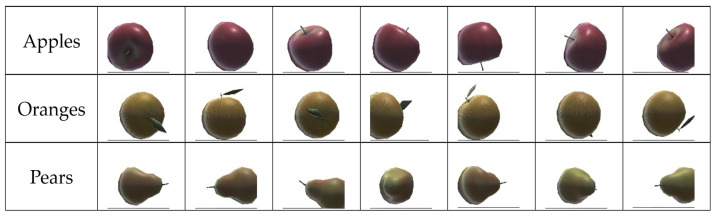
Illustrating the dataset for the Machine Learning process.

The dataset used to train and validate the CNN comprises images of fruit captured by the fruit distributor’s first camera. Figure 14 illustrates a subset of the dataset. Each class is composed of about one thousand fruit images.

**Figure 15:**
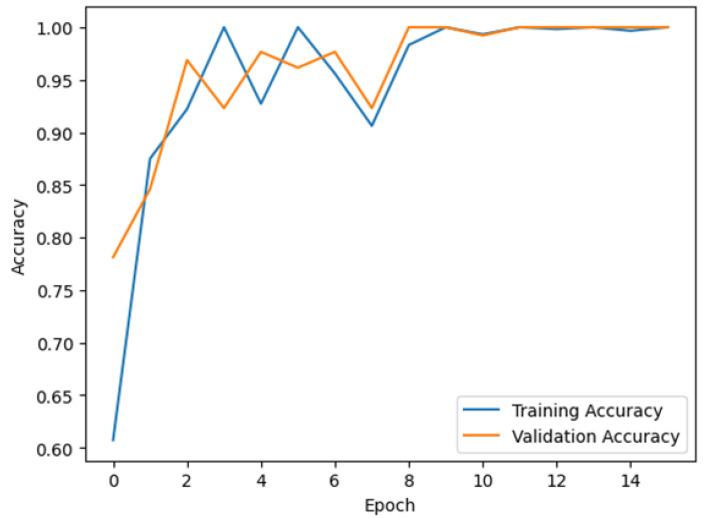
Evolution of the CNN model accuracies during the training and validation phases.

Figure 15 shows the evolution of the model’s accuracy during the training and validation phases. There are a few oddities that must be explained. Firstly, the fruit images were artificially created using the same textures into 3D objects representing fruits in a scene, in a virtual environment. As such, the resulting images lack the natural variability found in real-world data. Furthermore, even though the fruits were captured at different rotations and angles, the underlying texture patterns and features remain consistent across the images. These issues can make the task of distinguishing between different classes trivial and relatively easy for the model. Finally, the game scenario’s lighting, background, and environmental conditions remained unchanged and consistent. These issues led to a lack of variability in the data. As such, the training shows high accuracy early on and near-perfect validation.

Therefore, for more realistic results, using different object meshes for the same fruits, using varied textures, and changing the light conditions and backgrounds is necessary.

**Figure 16:**
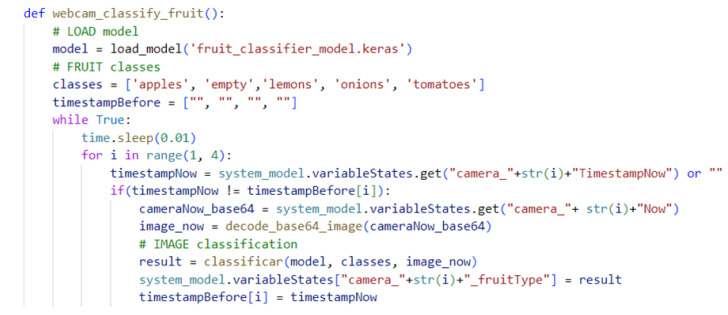
The script that loads the model and uses it to classify images for the corresponding fruits.

The trained and validated network is saved for later use in the back-end. The Python script that loads the network and classifies it is shown in Figure 16. The script is executed inside a thread that classifies every captured image of the corresponding fruit.

### 3.6. The Virtual PLC

Before ending this section, although it is not the focus of this work, it is important to briefly describe how the virtual PLC works.

The PLC is yet another virtual component of the architecture and platform developed. In this work, the virtual PLC (IEC PLC ST emulator) was developed with the features strictly necessary to carry out the proof of concept; the code was implemented in Python, inspired by previous works [50,51,52]. Its operation is similar to real PLCs, as illustrated in Figure 5.

**Figure 17:**
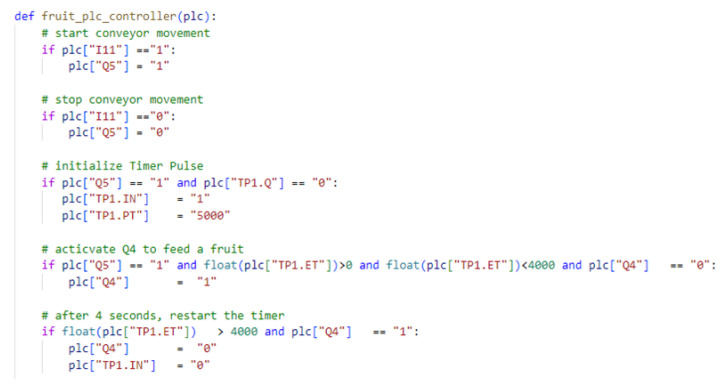
The Python code executed by the virtual PLC to control the feed of new fruits.

When the PLC starts running, the scan cycle is executed, in which the input variables are loaded into the PLC’s internal memory. Then, the rules for activating the outputs are executed and made available to the PLC outputs in the last step of the scan cycle (Figure 5). The Python rules for the PLC can be seen as equivalent to those used in Structured Text or Ladder Diagrams. A partial representation of the program that the PLC is executing is shown in Figure 17.

**Figure 18:**
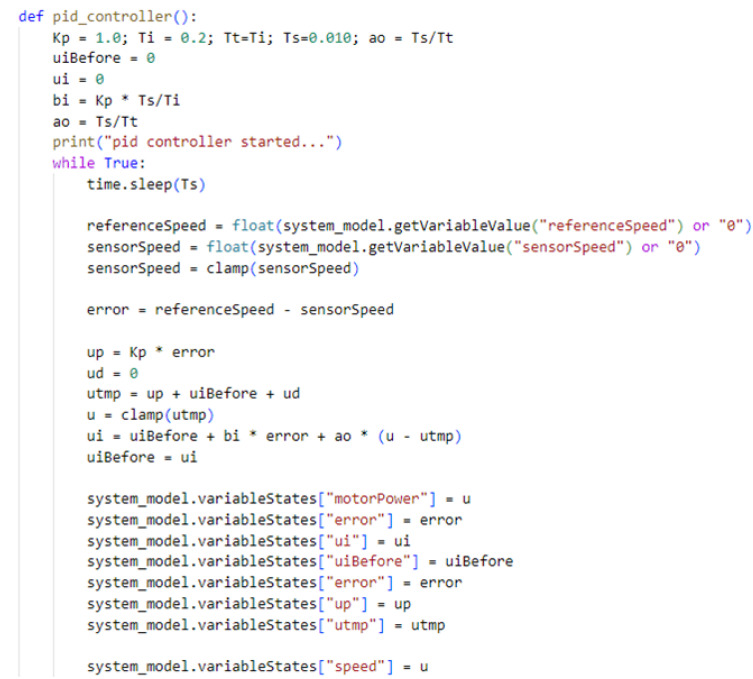
The Python code to control the conveyor power using a PI controller.

The PLC controller also executes the speed control code of the conveyor belt carrying the fruit, as shown in Figure 18. In this case, a Proportional–Integral (PI) controller, with an anti-windup term, is used. The controller compares the reference speed with the speed obtained from the camera-based virtual sensor described before. The error received is used to determine the control action to be taken, namely, the power applied to the motor.

## 4. Simulation Results and Reflection

**Figure 19:**
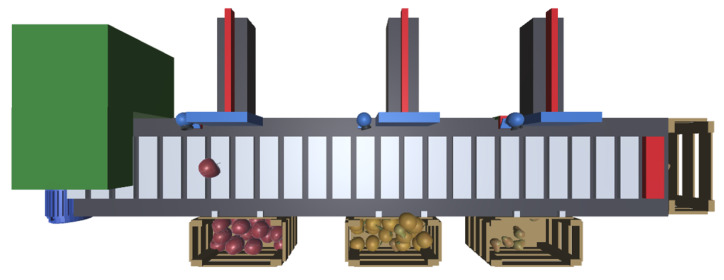
Illustration of the fruit splitter.

After the proof of concept was developed, it was put into operation. The conveyor started working and the fruits are placed on the conveyor belt. As described before, the sensor cameras detect the fruit. For each fruit identified in front of the respective box, the conveyor stops, and the cylinder is activated to move the fruit in the respective box. Two online videos, available at the addresses https://www.youtube.com/watch?v=0CznCIOeyCY, accessed on 18 November 2024 and https://www.youtube.com/watch?v=gJigT_ExIMY, accessed on 18 November 2024, elucidate how the system works. The project is also available at GitHub https://github.com/jrosas-AI/MDPI-paper. Figure 19 shows the system in operation with the fruit split into several boxes.

An interesting aspect of Figure 19 is that we can visually observe ConvNet’s performance. For example, if an orange is inside the apple box, that means the ConvNet provided a wrong classification.

**Figure 20:**
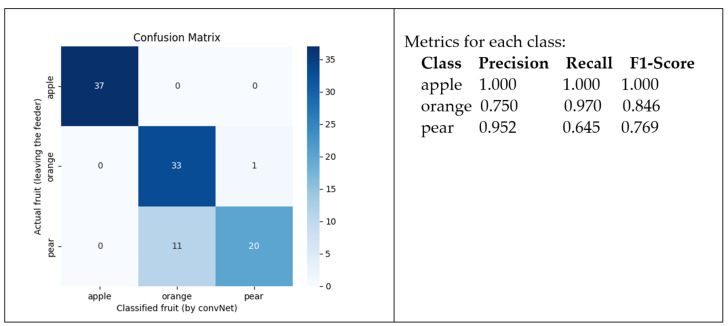
Confusion matrix for the trained ConvNet.

The confusion matrix that allows for evaluating the performance of the vision sensor is presented in Figure 20. As it turns out, despite the ML process converging on a solution with optimal quality, the network was failing to identify some fruits.

The apples and oranges were well identified. But some pears were classified as oranges. This is because, as shown in the dataset, some pear and some orange images seem to share some similarities in terms of color, texture, and shape. After verifying the game scenario, an issue related to illumination was found. The light for the scenario was adequate, but the bottom of the fruit captured by the cameras appeared darker. As the light for the scene was placed above, it caused shadows on the bottom of the fruit. Therefore, the captured images were not of sufficient quality. Although the ML process could converge, the classification of the fruit delivered by the feeder was sometimes wrong. Some pears were classified as oranges. On the other hand, apples were well classified, as they have different features from oranges and pears.

**Figure 21:**
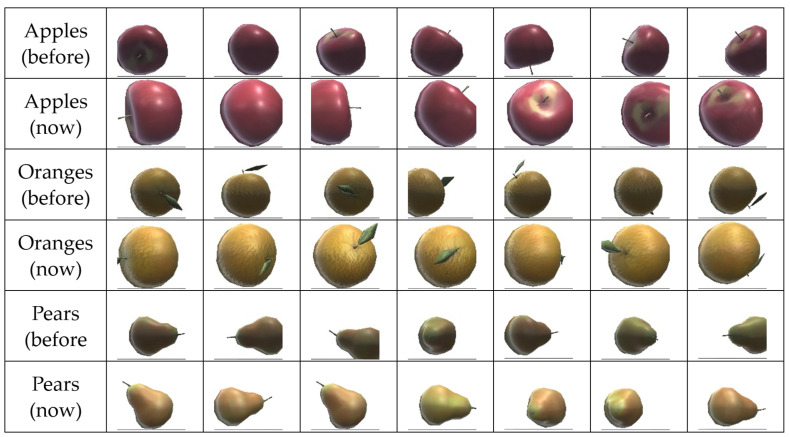
Comparing the previous and the improved fruit dataset.

Therefore, the next step was to solve the shadow effect below the fruit. For this, a light point was placed next to each camera so they could have a clearer view of the fruit captured. The result is illustrated in Figure 21. The images are also more detailed, allowing the algorithm to collect more distinctive patterns. We also proceeded to increase the size of the fruit slightly.

**Figure 22:**
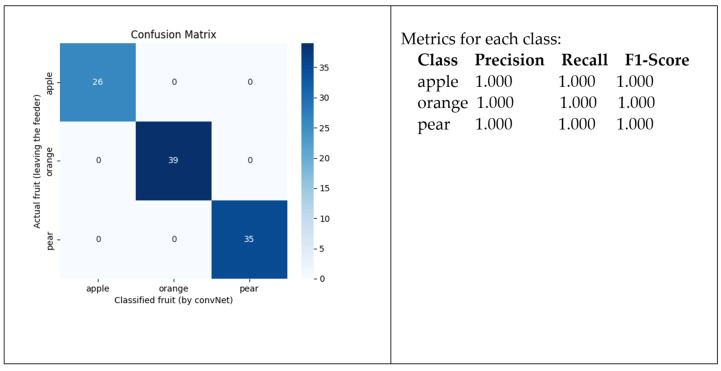
Confusion matrix for the trained ConvNet in the scene with improved light.

With this new dataset, the training phase was repeated. The results were similar to the previous ones, as the algorithm quickly converged. In terms of performance, the trained ConvNet expresses better quality, as shown in the corresponding confusion matrix and correspondent metrics presented in Figure 22.

Therefore, with better light conditions, the network successfully split all the fruits provided by the feeder. Given the apparent “absolute” success, the question is whether the model can also successfully classify fruit with more variety.

**Figure 23:**
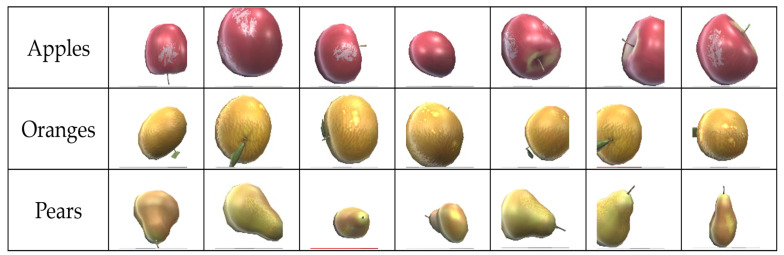
Dataset composed of fruit with variations in size, shape, and texture.

As such, another ML experiment was implemented comprising fruit of variable size and shape deformation and with slight changes in the texture, as shown in Figure 23.

**Figure 24:**
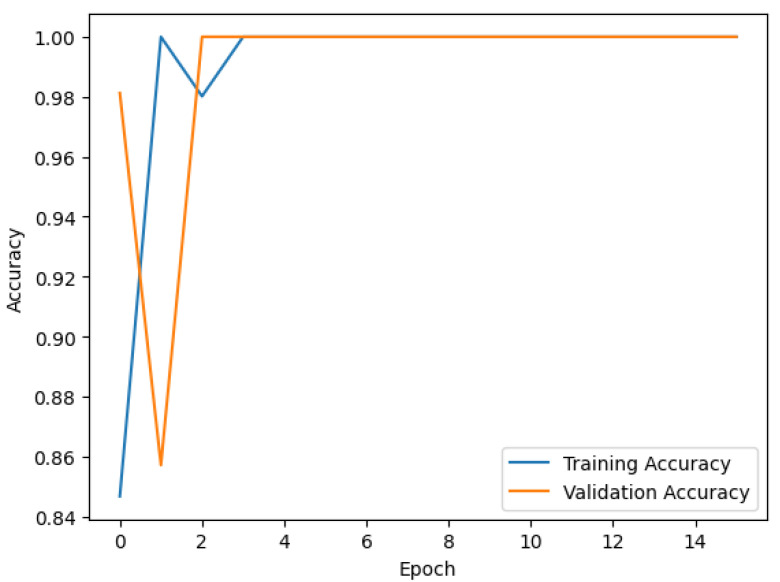
Evolution of the model accuracy for the dataset with more fruit variability.

**Figure 25:**
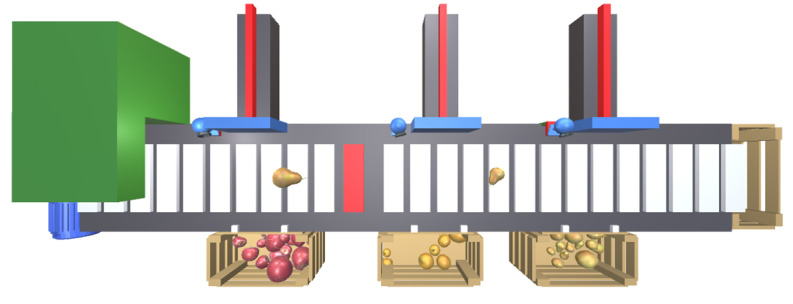
Fruit splitter with 50% variation in fruit size.

The ML process also achieved a high success rate with this new dataset, as shown in Figure 24. However, since the model achieved near-perfect accuracy and very low loss early in the training, there might be a risk of overfitting. Therefore, to confirm generalization, it was recommended to test the model on a separate dataset, which the model did not see during training or validation. As such, the feeder behavior was modified to partially develop this test. Over the fruit that was already subjected to deformation and texture change, the feeder delivered fruit with a 50% size variation. The simulation was executed again, as illustrated in Figure 25.

**Figure 26:**
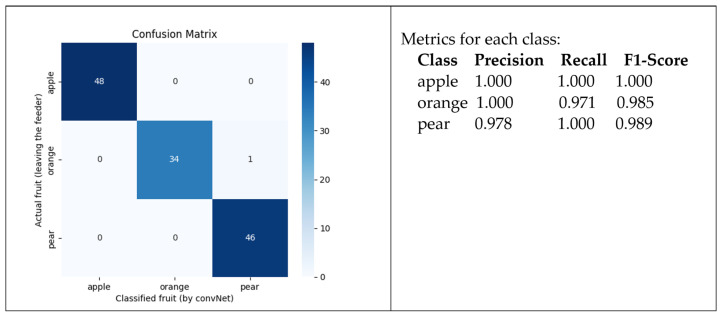
Confusion matrix for training with variable fruit size.

This time, of the 129 pieces of fruit that were classified, one orange was classified as a pear. Figure 26 shows the corresponding confusion matrix and metrics. The model’s performance is still outstanding, considering that a 50% variation in the fruit sometimes resulted in more extreme fruit sizes. Through this last experience, we can consider that developing a platform based on a game engine offers many possibilities for generating datasets for ML algorithms [41,53,54,55,56,57,58].

The PLC and its program for controlling the fruit distribution system also work correctly. The PID controller also works correctly, and this was possible because we could successfully use the camera to observe the movement of the red strip on the conveyor belt and calculate its speed.

Considering the results obtained, the proposed approach to virtualizing sensors seems to work relatively well, a conjecture which requires further research. Upon achieving the final system, the perception is that the virtualization process is greatly facilitated through game engines. The approach is intrinsically simple and easy to replicate for other virtual scenarios when creating other virtual environments. Simplicity was one of the main goals of the proposed approach. The authors believe that the best solutions are precisely the ones that are simple and work well. Such is the case of the proposed six-step approach, which allows this virtualization to be carried out straightforwardly.

The proposed approach can be used to create virtual environments for training and teaching, for systems simulation, and in testing solutions for control and automation, among others.

As mentioned before, game-based environments allow for the generation of datasets for ML, which would be more difficult otherwise. In a real context, we would need a fruit distribution station or, at the very least, a lot of fruit to photograph.

As a limitation, some systems require a real-time clock shared between the various components and robust synchronization mechanisms. These aspects were not relevant to the proof of concept presented, but they are essential in simulations with stricter real-time requirements.

Finally, the convolutional neural network (CNN) used in this simulator reveals good robustness, as it had very good performance even in situations where the images are incomplete and have some imperfections. This robustness feature is crucial in practical applications, where data from the real world can have high variability, imperfections, noise, etc.

## 5. Conclusions

This research proposed a straightforward approach to specifying and developing virtual sensor models to simulate virtual environments in the Industry 4.0 context.

The approach consists of six steps, which include the analysis of a sensor, the specification of its behavior, the definition of its interfaces, its development, its validation, and finally, its integration and commissioning in the virtual environment.

The developed proof of concept comprised modeling a fruit distribution system in a virtual world using a game engine. It involved the development of a platform composed of several modules, including a PLC and a ConvNet. The PLC module integrates logic control and a PID controller. The platform controls a conveyor belt with a feeder for providing fruit. The conveyor has three exit points, each dedicated to a specific fruit. Each point has a pneumatic cylinder, a binary sensor to detect the presence of the fruit, and a camera that captures images and sends them to the ConvNet module. If the image matches the fruit destined for the respective exit point, the cylinder pushes the fruit into the corresponding box.

The results allowed us to conclude that the proposed approach can be applied intuitively to various types of sensors, including those of higher complexity, like vision sensors. The vision sensors are represented by cameras that capture images of fruit and send them via web requests to a Python module. This module uses a robust convolutional neural network (ConvNet) to identify whether each image corresponds to an apple, orange, or pear.

One of the potential uses of this approach is in education and training. Thus, as a future work, creating virtual reality models is proposed to improve the impact of learning during classes.

As a future work, the proposed method should be refined and extended to more types of sensors. A similar approach for virtualizing actuators, controllers, and other components of a virtual system is also worthwhile.

The six-step sensor virtualization approach was successfully applied. However, being an essentially experimental approach, several aspects for improvement were identified. These improvements include using formal models and mechanisms to formalize each step of the approach.

## Figures and Tables

**Table 1:** Comparative analysis of main features for different simulators.

Simulator/Features	Our Simulator	Factory I/O	Machines Simulator	CodeSys
Build your own 3D scenario	yes	yes, but limited to a library of a set of components	yes, but limited to a library of a set of components	yes, but limited to a library of a set of components
Digital and analog I/O	yes, via Python	yes	yes	yes
Interaction with real PLCs	no	yes	yes	yes
Interaction with virtual PLCs	yes: IEC PLC ST emulator	yes: built-in control I/O, Siemens S7-PLCSIM, WinSPS-S7, CodeSys	yes: Siemens S7-PLCSIM, SIMIT, EasyPLC, CodeSys	yes: Siemens, Schneider Electric, Beckhoff, WAGO, and more
Interaction with microcontrollers	yes, via Python	yes, via Modbus TCP/IP, OPC-UA, custom drivers	yes, via Modbus TCP/IP, OPC-UA, custom drivers	yes: Codesys control, Modbus TCP/IP, OPC-UA, custom drivers
Supported languages and tools	Unity 3D, Python, IEC PLC ST emulator	5 IEC PLC languages (LD, IL, SFC, FBD, ST)	5 IEC PLC languages (LD, IL, SFC, FBD, ST)	5 IEC PLC languages (LD, IL, SFC, FBD, ST)
Integration of advanced approaches (AI/ML, optimization, big data, IOT, web services)	yes, via API	yes, via API	yes, via API	yes, via API
Freeware	yes	no	no	no

**Table 2:** Ways to implement behavioral mapping between real sensors and their simulation models with game engine features.

Type	How	Game Engine Components
Contact	An object touches the sensor.	Colliders and scripts
Proximity	An object approaches the sensor.	Raycast will detect the nearest object, the overlapping sphere, and the scripts.
LIDAR; ultrasound	Obtaining the distance from a sensor to an object.	Raycast and scripts.
Object recognition	When a camera observes an object, it grabs a picture/texture.	Capture the picture from the camera; use external APIs for feature identification, e.g., OpenCV and ML.

**Table 3:** Application of the virtualization approach to a proximity sensor.

Phase	Description
1	When an object approaches the sensor, a collider can be used to capture the event of passing through this zone.
2	If a fruit enters the collider zone, set the status variable to “True”. If it leaves, set it to “False”.
3	The definition of the interface for the sensor. In this case, it could be, for example, http://hostaddress:port/getVariableValue?Name=Sensor_1.
4	Implement the sensor model, including its behavior. To perform this, create a script with methods that capture the fruit’s entry/exit event in the collider. Set the state of the sensor variable to the corresponding value.
5	Carry out tests within the virtual environment. Check that the state variable changes to True when a fruit crosses the collider zone. If any differences are detected between the current behavior and the desired one, return to step 1.
6	Integrate the sensor into the virtual environment. Considering the game engine, add an object to the scene hierarchy representing the virtual environment.

**Table 4:** Application of the virtualization approach for measuring the conveyor speed.

Phase	Description
1	Place the camera above the conveyor to observe its movement. A visible red strip on the conveyor belt is used as a reference for measuring speed.
2	Take successive image captures over time. Each image obtained is sent to the back-end. At this point, the OpenCV library is used to measure the position of the red strip against the left side of the captured image. Accumulate the last ten positions in an array with the positions and timestamp. Use a regression algorithm to estimate the current speed.
3	As before, an interface is defined to access the variable’s value that provides the current speed. As the state variables are stored in the back-end memory, we use the address http://localhost:8089/getVariableValue?Name=\sensorSpeed.
4	Implement the sensor by creating a script with methods that capture the strip movement on the conveyor.
5	Carry out tests in the game scenario. Check that the estimated speed in the sensorSpeed status variable corresponds to the movement perceived on the carpet.
6	Integrate the sensor into the virtual environment. Considering the game engine, add an object to the scene hierarchy representing the virtual environment.

**Table 5:** Application of the virtualization approach to a fruit identification sensor.

Phase	Description
1	Once the proximity sensor has been activated, capture an image from the camera observing the fruit being transported on the conveyor.
2	The image obtained is sent to the server, making it available to ConvNet to identify the fruit. The result of the identification is placed in a state variable with the name, for example, camera_1_fruitType, whose value could be “apple”, “orange”, or “pear”.
3	As before, an interface is defined. Since the state variables are stored in the back-end memory, we use the address http://localhost:8089/getVariableValue?Name=camera_1_fruitType.
4	Implement the sensor by creating a script with methods that use the camera to capture the fruit image, store it in a C# class property, and make it available to be sent to the back-end.
5	Carry out tests in the game scenario. Check that the state variable changes to True when a fruit crosses the collider zone.
6	Integrate the sensor into the virtual environment. Considering the game engine, add an object to the scene hierarchy representing the virtual environment.

## Data Availability

Two online videos demonstrating how the system works are available at https://www.youtube.com/watch?v=0CznCIOeyCY, accessed on 18 November 2024 and https://www.youtube.com/watch?v=gJigT_ExIMY, accessed on 18 November 2024. The project source-code and results are available at https://github.com/jrosas-AI/MDPI-paper.

## Abbreviations

3D	Three Dimensional
AAS	Asset Administration Shell
AI	Artificial Intelligence
API	Application Programming Interface
CNN	Convolutional Neural Network
CPS	Cyber-Physical Systems
DT	Digital Twins
GE	Game Engine
IIot	Internet of Industrial Things
IoT	Internet of Things
LiDAR	Light Detection and Ranging
ML	Machine Learning
PID	Proportional–Integral–Derivative
PLC	Programmable Logic Controller
VAR	Virtual and Augmented Reality
VR	Virtual Reality
